# Supplementary material for: Deep mutational scanning of EccD3 reveals the molecular basis of its essentiality in the mycobacterium ESX secretion system
Source: bioRxiv. 2024 Aug 24:2024.08.23.609456. Preprint. [Version 1] doi: 10.1101/2024.08.23.609456 (PMC11370616; doi:10.1101/2024.08.23.609456)
Supplement: Supplement 1 [file NIHPP2024.08.23.609456v1-supplement-1.pdf]

bioRxiv preprint doi: https://doi.org/10.1101/2024.08.23.609456; this version posted August 24, 2024. The copyright holder for this preprint (which was not certified by peer review) is the author/funder, who has granted bioRxiv a license to display the preprint in perpetuity. It is made available under aCC-BY 4.0 International license.

Figure S1.

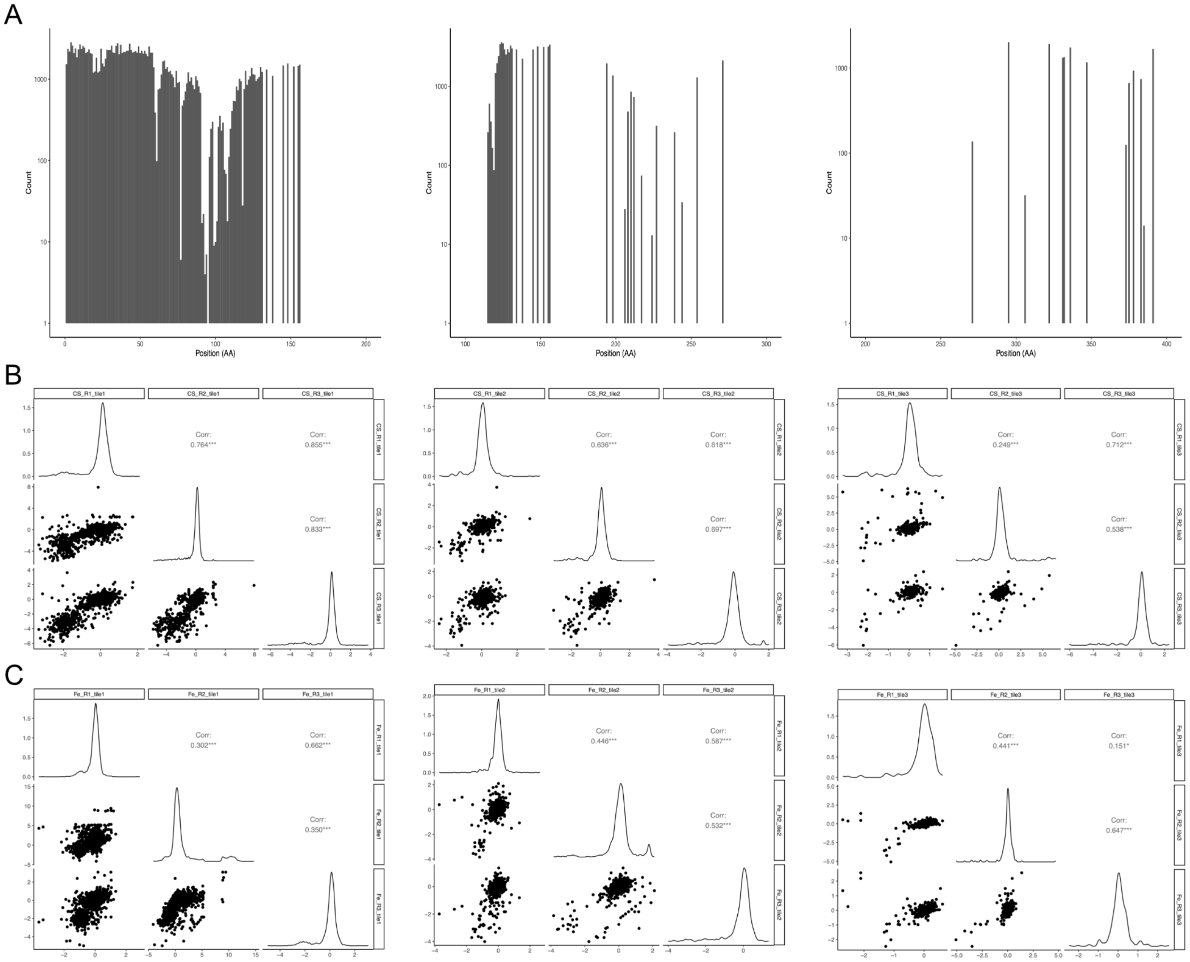

EccD$_3$ library scores and QC.

A) Overall distribution of library counts (Y-axis) per position (X-axis) for each tile. B) Cross-correlations between EccD$_3$ iron-deficient screen replicates. Each set of plots represents cross-correlations for each tile. Pearson correlation coefficients above diagonal, histograms of scores for replicates on the diagonal, and dot plots with variants below the diagonal. C) Cross-correlations between EccD$_3$ iron-sufficient screen replicates, same as B.

bioRxiv preprint doi: https://doi.org/10.1101/2024.08.23.609456; this version posted August 24, 2024. The copyright holder for this preprint (which was not certified by peer review) is the author/funder, who has granted bioRxiv a license to display the preprint in perpetuity. It is made available under aCC-BY 4.0 International license.

Figure S2.

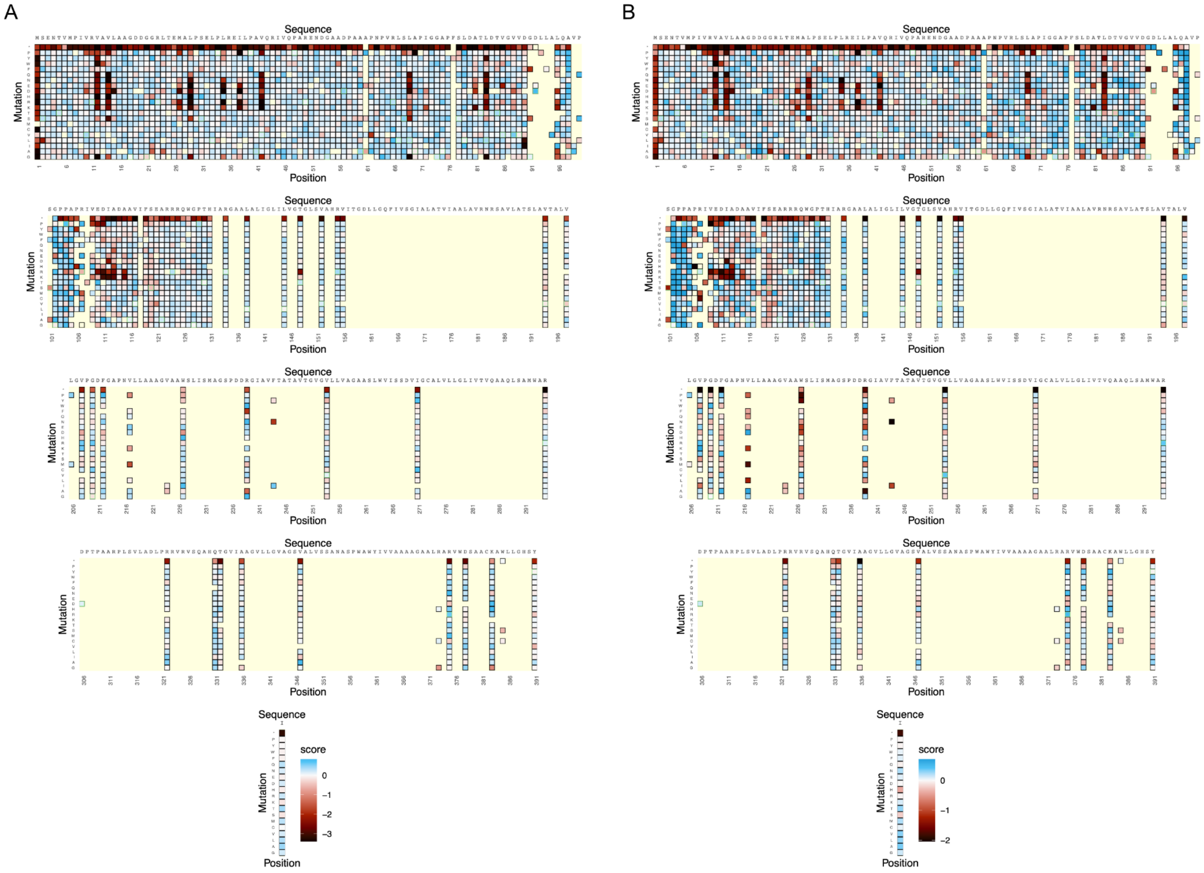

EccD$_3$ heatmaps

A) Heatmap of the EccD$_3$ functional scores from the iron-deficient growth condition. The WT sequence, domain organization, and cartoon secondary structure representation of EccD$_3$ are shown above each section of the heatmap. The variant identity is indicated on the y-axis, and the residue position is indicated on the x-axis. WT-synonymous substitutions are outlined in green and positions not included in the library are light yellow. B) Heatmap of the EccD$_3$ functional scores from the iron-sufficient growth condition.

bioRxiv preprint doi: https://doi.org/10.1101/2024.08.23.609456; this version posted August 24, 2024. The copyright holder for this preprint (which was not certified by peer review) is the author/funder, who has granted bioRxiv a license to display the preprint in perpetuity. It is made available under aCC-BY 4.0 International license.

Supplemental Table 1.

Pre-optimization			
	Dilution 1, cfu	Dilution 2, cfu	Dilution 3, cfu
100ng	32	3	0
500ng	67	6	0
1µg	123	13	3
2µg	155	49	7
**Post-optimization**			
	Dilution 1, cfu	Dilution 2, cfu	Dilution 3, cfu
100ng	312	33	1
500ng	360	63	1
1µg	647	81	2
2µg	--	112	10
Library 1µg	--	285	24
